# Isolation, Characterization and Growth-Promoting Properties of Phosphate-Solubilizing Bacteria (PSBs) Derived from Peach Tree Rhizosphere

**DOI:** 10.3390/microorganisms13040718

**Published:** 2025-03-23

**Authors:** Zixuan Li, Junyan Li, Guangyuan Liu, Yanyan Li, Xuelian Wu, Jiahui Liang, Zhe Wang, Qiuju Chen, Futian Peng

**Affiliations:** College of Horticultural Science and Engineering, Shandong Agricultural University, Tai’an 271018, China; lllizx@163.com (Z.L.); ljyxxgz@163.com (J.L.); lgyliu2001@163.com (G.L.); liyanyan2575@163.com (Y.L.); xuelianwu229@163.com (X.W.); liangljh@163.com (J.L.); wzhelaiwu@163.com (Z.W.); lmchen.ju@163.com (Q.C.)

**Keywords:** phosphate-solubilizing capacity, iron carriers, biofilm, potting experiment, phosphate solubilization index

## Abstract

Microbial fertilizers have a significant role in promoting plant growth, resistance to environmental stresses, and soil remediation. Microbial fertilizers are mainly composed of beneficial microorganisms that contain specific functions. Focusing on the peach tree rhizosphere region, this study aimed to isolate and screen bacteria with efficient phosphate-solubilizing capacity for application in microbial fertilizers, as well as to dig deeper into the other properties of the strains to further explore the roles of these phosphate-solubilizing bacteria (PSBs) in terms of plant growth in order to provide valuable microbial resources for microbial fertilizer development. By collecting soil samples from peach tree rhizospheres, we initially screened 86 PSB strains using the plate method and determined the phosphate-solubilizing capacity (ranged from 0 to 14 μg/mL). Afterwards, 51 strains with strong phosphate-solubilizing capacity were selected for molecular identification; the strains belonged to 12 genera, with *Bacillus* and *Burkholderia* accounting for the majority. Concurrent evaluation of iron carriers and indoleacetic-3-acid (IAA) production capabilities identified strain WPD85 as exhibiting dual functionality with strong performance in both parameters. Subsequently, we combined the analysis of phosphate-solubilizing capacity and growth-promoting properties to select eight strains of PSBs; characterized them physiologically, biochemically, and molecularly; determined the biofilm-forming capacity; and conducted potting experiments. Notably, strain WPD103 exhibited exceptional biofilm-forming capacity (OD_595_ = 1.09). Of particular interest, strain WPD16 demonstrated both an elevated inorganic phosphate solubilization index (D/d = 2.99) and remarkable iron carriers production capacity, while peach seedlings treated with WPD16 exhibited 119% enhancement in plant height increment compared to the control. This study enhances our understanding of PSB traits and identifies *Burkholderia* sp. WPD16 as a strategic candidate for developing targeted microbial fertilizers, offering a sustainable solution to reduce reliance on chemical inputs in orchard management.

## 1. Introduction

Phosphorus (P) plays a very important role in the entire life cycle of plants, being one of the essential elements for plant growth and metabolism [1]. The effectiveness of phosphorus in natural soils is frequently constrained by its low solubility and ease of immobilization, which impedes the capacity of plants to absorb and utilize it directly [2]. In the extensive domain of agricultural production, the efficient utilization of soil phosphorus has been identified as a pivotal element in constraining the enhancement of crop yield and quality [3]. Rhizosphere microorganisms play an important role in regulating the soil phosphorus cycle and are able to convert insoluble P in the soil into plant-absorbable forms, thus improving the availability of phosphorus to plants [4]. At the same time, the development of microbial fertilizer can be called a milestone in the history of fertilizer development. Microbial fertilizers are not only environmentally friendly and renewable; they also promote sustainable agriculture. Microbial fertilizers help to restore soil ecosystems, and in turn, they promote nutrient uptake, regulate crop growth, and increase crop resistance to biotic and abiotic stresses [5,6].

In recent years, with the rapid development of microbiology, molecular biology, and biotechnology, research into the use of microbial phosphate-solubilizing capacity to improve soil phosphorus nutrient status has attracted increasing attention. Researchers have isolated a variety of microorganisms from different ecosystems that have the ability to detoxify phosphates. These microorganisms secrete organic acids, phosphatases, and other substances, thereby increasing the effective phosphorus content of the soil and improving the soil environment to ensure the normal growth and development of plants [7]. Some studies have reported that PSB inoculation significantly increased the yield of maize, wheat, and rice crops, as well as increased effective phosphorus content, enzyme activity, and soil fertility in the crops [8,9,10,11,12,13]. Furthermore, it was found that inoculation of PSBs could increase the content of soluble sugar and soluble protein in apple, while also increasing the P content in the soil of apple seedlings and the uptake of P by apple seedlings to affect the growth and development of plants [14,15,16,17]. The above studies have shown that the application of PSBs promotes crop phosphorus uptake, crop growth, and yield; therefore, PSBs have great potential to be applied in the development of microbial fertilizers. However, most of the current studies on PSBs are based on crop sources [18], and correspondingly, there are relatively few studies on PSB microorganisms from fruit trees. While PSBs from crops have been extensively studied, their counterparts in fruit-tree ecosystems remain underexplored, limiting the development of specialized biofertilizers for orchards. The diverse sources of microbial resources used as microbial fertilizers result in microbial fertilizers that are not targeted to maximize their usefulness. Therefore, it is imperative to explore the microbial resources applied to microbial fertilizers for developing ecosystem-specific biofertilizer formulation, enhancing nutrient use efficiency in perennial crops, and reducing dependence of chemical fertilizer.

The peach tree is an important fruit tree cash crop, and China is the world’s largest peach producer. The peach industry gross output value of nearly CNY 100 billion and its more than ten million production, distribution, and retail employees promote the countryside’s industry prosperity, so farmers represent a rich main economic pillar [19]. In addition, the peach also has a unique landscape value and cultural connotation, contributes to the construction of a beautiful countryside, promotes industrial integration, and carries forward farming culture as an important industrial support. However, intensive fertilization practices in peach cultivation have led to soil degradation and phosphorus fixation, threatening long-term sustainability. Therefore, this study aims to address this issue by isolating and characterizing PSBs specifically from peach tree rhizospheres, with a focus on their dual roles in phosphate solubilization and plant growth promotion.

In this study, we screened efficient PSBs from the rhizosphere of peach tree soil by the plate method of isolation and determined its various capacities, carried out identification and biological characterization of them, and performed a potted experiment to study in depth the phosphate-solubilizing microorganisms in the soil sourced from fruit trees, make the source of PSBs more complete, and broaden the scope of application of the phosphate-solubilizing bacteria. At the same time, the validation of PSBs from fruit trees provides more resources for the research and development of microbial fertilizers in order to develop microbial fertilizers dedicated to the cultivation of fruit trees. This will not only greatly improve the utilization rate of microbial fertilizer and make the application of microbial fertilizer more scientific and efficient; it will also ensure the precision of agriculture applications, promote soil health restoration initiatives, and develop circular agricultural systems for a more healthy and sustainable development of agriculture.

## 2. Materials and Methods

### 2.1. Sample Collection and Isolation of PSBs

Soil samples were collected from the rhizosphere of peach trees in orchards under two modes of fertilization in Feicheng, Tai’an City, Shandong Province, China. One mode is to apply bag-controlled release fertilizer (BCRF: N:P_2_O_5_:K_2_O = 2:1:2) year-round, and the other is to apply compound fertilizer according to the traditional mode year-round. For each fertilization mode, we selected 5 trees and collected the soil located around the peach tree rhizosphere in the soil layer 0–20 cm and mixed it. The soil was then sieved through a 2 mm sieve, placed into an aseptic self-sealing bag, and stored in a refrigerator set at 4 °C.

Five grams of soil was suspended in 10 mL sterile water and shaken at 200 rpm (28 °C, 2 h) (Shaker: MQT-60P, Minquan Instrument, Shanghai, China). Following this, the soil suspension was prepared according to a 10-fold dilution gradient [20]. The soil suspension (with a dilution gradient of 10^−4^, 10^−5^ and 10^−6^) was then pipetted into a volume of 200 μL, which was then spread on the PVK solid medium (glucose, 10 g/L; (NH_4_)_2_SO_4_, 0.5 g/L; NaCl, 0.3 g/L; MgSO_4_, 0.3 g/L; K_2_SO_4_, 0.3 g/L; FeSO_4_, 0.03 g/L; MnSO4, 0.03 g/L; Ca_3_(PO_4_)_2_, 5 g/L; agar, 15 g/L; pH 7.0–7.5) in an even layer and placed into an inverted position to incubate at a temperature of 28 °C. Single colonies exhibiting vigorous growth were meticulously selected and purified through multiple streak isolations until a single colony was obtained. These colonies were then stored in a refrigerator maintained at a temperature of 4 °C. The purified strains were stored in a glycerol stock solution (50%) that was placed at −80 °C for subsequent experimental procedures.

### 2.2. Assay of Phosphate-Solubilizing Capacity

The dephosphorylated strains obtained by isolation and purification were inoculated into centrifuge tubes containing 10 mL of LB liquid medium and placed in a thermostatic shaker at 28 °C and 200 r/min for overnight incubation. A quantity of 10 μL of the seed solution was carefully pipetted and inoculated into NBRIP solid medium (glucose, 10 g/L; (NH_4_)_2_SO_4_, 0.1 g/L; MgSO_4_·7H_2_0, 0.25 g/L; MgCl_2_, 5 g/L; KCl, 0.2 g/L; Ca_3_(PO_4_)_2_, 5 g/L; agar, 15 g/L; pH 7.2 ± 0.2) [21]. The diameter of the halo zone (D) and the diameter of the colony (d) were then measured after inverted incubation at 28 °C for 3 days. Finally, the D/d value was calculated. Concurrently, the seed solution was inoculated into NBRIP liquid medium at an inoculum of 2% (*v*/*v*). A 2% (*v*/*v*) sterile water solution was utilized as a blank control in lieu of the seed solution, which was repeated This procedure was repeated on three occasions, and the samples were then incubated in a thermostatic shaker at 28 °C and 200 r/min. After a period of 6 days, the samples were subjected to centrifugation (Centrifuge: ST 8R, Thermo Scientific), and the soluble-phosphate production in the upper layer was measured by means of a molybdenum antimony antimicrobial colorimetric assay (Spectrophotometer: TU-1950, PERSEE, Beijing, China) [22].

### 2.3. Characterization of PSB Isolates

Direct observation of the eight dominant strains was performed after screening, including convexity, color, transparency, verge, and shape. The strains were subjected to Gram’s staining, and an additional 4% KOH supplementation test was performed. Simultaneously, an examination was conducted of the physiological and biochemical characteristics of the strains. This was undertaken using a range of characteristic tests, including sugar fermentation, starch hydrolysis, catalase, methyl red test, citrate utilization test, antibiotic susceptibility test, lipase, urease, and so forth. Details of this work can be found in the supplementary File S1 [23].

For the 51 strains of PSBs, after rescreening, 16SrRNA sequence analysis was performed. The DNA of the test strains was extracted using a bacterial genomic DNA extraction kit(CW0552S, CWBIO, Jiangsu, China) and amplified by PCR(T30, LongGene, Hangzhou, China) using bacterial universal primers, 27F and 1492R [24]. The PCR reaction system comprises the following components: genomic DNA (0.5 μL), 10 × Buffer with Mg^2+^ (2.5 μL), dNTP (1 μL), RNA polymerase (0.2 μL), 10 μmol/L upstream and downstream primers (0.5 μL each), and double-distilled water (25 μL). The PCR conditions are as follows: 94 °C pre-denaturation 45 s; 55 °C reversion 45 s; 72 °C extended 1 min, a total of 30 cycles; 72 °C repair extension 10 min; and terminate the reaction at 4 °C. The PCR amplification products were sent to Bioengineering (Shanghai) Co., Ltd., for sequencing. The sequencing results were compared on NCBI, and the phylogenetic tree was constructed using the software MEGA 11 [25].

### 2.4. Determination of the Growth-Promoting Ability of PSBs

The experiment was conducted in order to ascertain the IAA production capacity of the strain. The strain was inoculated into LB liquid medium (with 0.5 g·L^−1^ L-tryptophan added) according to an inoculum amount of 2%. The inoculated medium was then placed in a 28 °C, 200 r/min shaker culture for 5 days. The bacterial suspension was then subjected to centrifugation in order to obtain the upper layer. Salkowski’s reagent was then mixed with this upper layer. The ratio of Salkowski’s reagent to bacterial suspension was 1:2. The color-development stage was then conducted at room temperature in the dark for 30 min. The color was then observed at 530 nm. Following the 30-minute color-development stage, the color was observed at 530 nm to determine whether it was red. The yield of IAA at 530 nm was then determined [26].

The production capacity of iron carriers is determined by the following procedure. The strain is first inoculated onto a CAS solid medium. This is then incubated at 28 °C for a period of seven days. At this point, the presence of a yellow halo around the colony should be observed [27,28].

### 2.5. Determination of Biofilm Formation Ability of PSBs

Biofilm formation was quantified using 96-well polystyrene microtiter plates. Each well received 100 μL of culture medium inoculated with 10 μL bacterial suspension, followed by 48 h of incubation at 37 °C. Post-incubation, adherent cells were washed thrice with 200 μL sterile PBS and fixed with 100 μL methanol (15 min at RT). After air-drying, biofilms were stained with 1% crystal violet (100 μL/well, 5 min). Excess stain was removed by rinsing under running water, and plates were inverted for moisture removal prior to 37 °C oven drying. Bound dye was solubilized with 100 μL 33% glacial acetic acid (30 min at 37 °C). Optical density at 595 nm was measured using a CMax Plus microplate reader (Molecular Devices, Silicon Valley, America). All experiments included triplicate wells per strain, with uninoculated medium as negative control. The cutoff value (Dc) was defined as twice that of the negative control, OD595 [29].

### 2.6. Effect of PSBs on Growth of Peach Seedling

The 2% inoculum of the strain was inoculated in LB liquid medium, placed in a constant temperature oscillator at 28 °C and 200 r/min, and then incubated for 2 days before being configured into a suspension of 4 × 10^8^ CFU·mL^−1^. One-year live hickory seedlings were used as test materials and planted in pots of 15 × 15 cm, with nutrient soil as the cultivation substrate. After cultivation for a period of time, peach seedlings with consistent growth conditions were selected for treatment, and each seedling was treated with 30 mL of root irrigation every 7 days, for a total of 4 times. Meanwhile, fresh water was used to treat as a control. Plant height and stem thickness were recorded before the first treatment. After 40 days of treatment, plant height and stem thickness were measured, and root conformation was scanned and analyzed using the professional version of Win RHIZO 2017a (Rgent Instruments Inc., Quebec, QC, Canada) root analysis software (resolution of 300 dpi), and the parameters were set with reference to the instructions of the software.

### 2.7. Data Analysis

Excel 2011 was used for data sorting, and IBM SPSS Statistics 22 was used for performing one-way ANOVA (Shapiro–Wilk) and significance analysis (Tukey’s b), with at least 3 replications for each set of data, and the difference significance was defined as *p* < 0.05. GraphPad Prism 9 and MEGA 11 software were used for mapping, and the iTol website was used for evolutionary tree landscaping. The images were merged using Adobe Photoshop 2019.

## 3. Results

### 3.1. Isolation and Identification of PSBs

**Figure 1:**
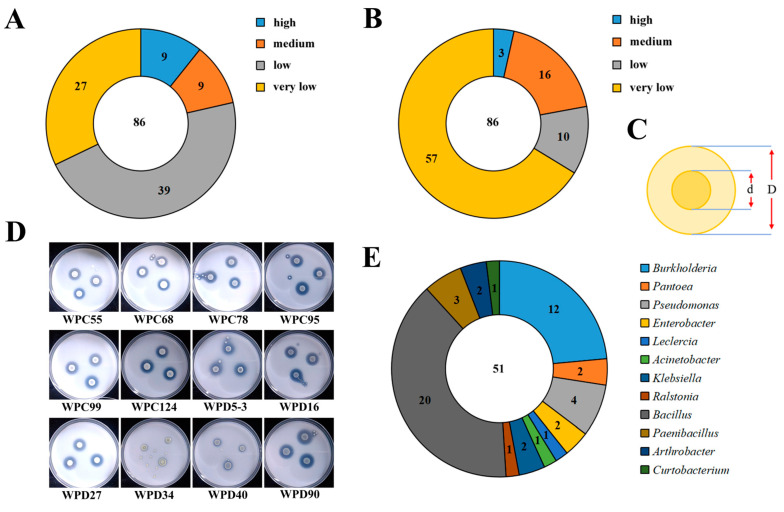
Preliminary screening and identification of PSBs. (**A**) Number of PSBs in liquid medium for soluble phosphorus content with different solubilization potential (high ≥ 13 mg/L; 10 mg/L ≤ medium < 13 mg/L; 7 mg/L ≤ low < 10 mg/L; very low < 7 mg/L). (**B**) Number of PSBs in solid medium for phosphate solubilization index with different solubilization potential (high: D/d ≥ 3; medium: 2 ≤ D/d < 3; low: 1.5 ≤ D/d < 2; very low: D/d < 1.5). (**C**) Phosphate solubilization index (D/d) pattern (halo zone diameter/strain diameter). (**D**) Pictures of some halo zone of PSBs. (**E**) Number of PSBs in each genus after rescreening.

**Figure 2:**
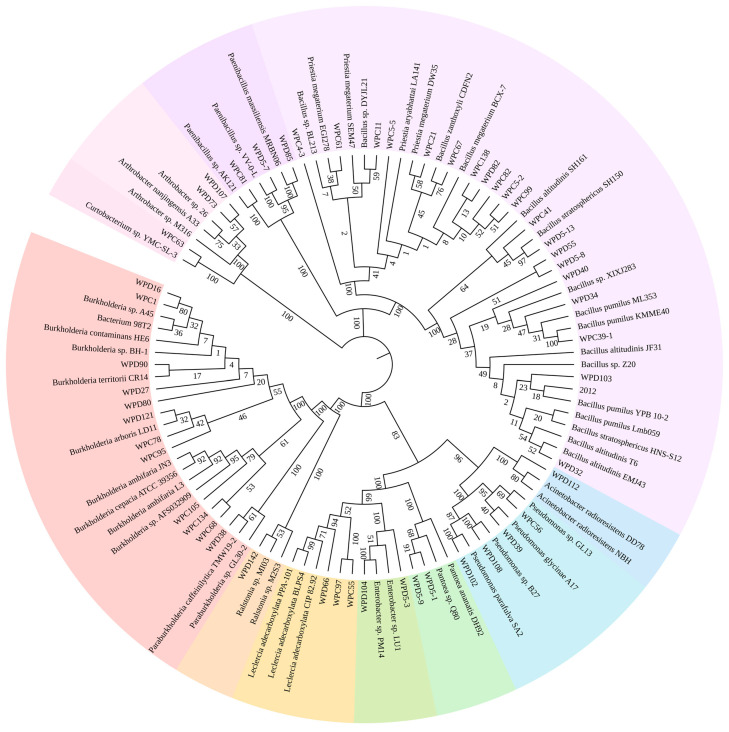
Phylogenetic tree based on 16SrRNA sequences.

Our study used NBRIP medium containing Ca_3_(PO_4_)_2_ as the sole P source for the screening strategy to isolate PSB colonies. A total of 86 PSBs were isolated and purified from peach tree rhizosphere soil under two fertilization regimes. Through measuring soluble phosphorus content in liquid media and phosphate solubilization index on solid media, we found that nine strains have high soluble phosphorus content in liquid media, while three strains have high phosphate solubilization index on solid media (Figure 1A). Based on both phosphate-solubilizing capabilities, 51 high-performance strains were selected for 16S rRNA sequencing analysis. The results revealed that these 51 strains belonged to 12 genera, with *Bacillus* (20 strains) and *Burkholderia* (12 strains) being the predominant genera (Figure 1E and Figure 2).

### 3.2. Determination of Growth-Promoting Ability of PSBs

**Figure 3:**
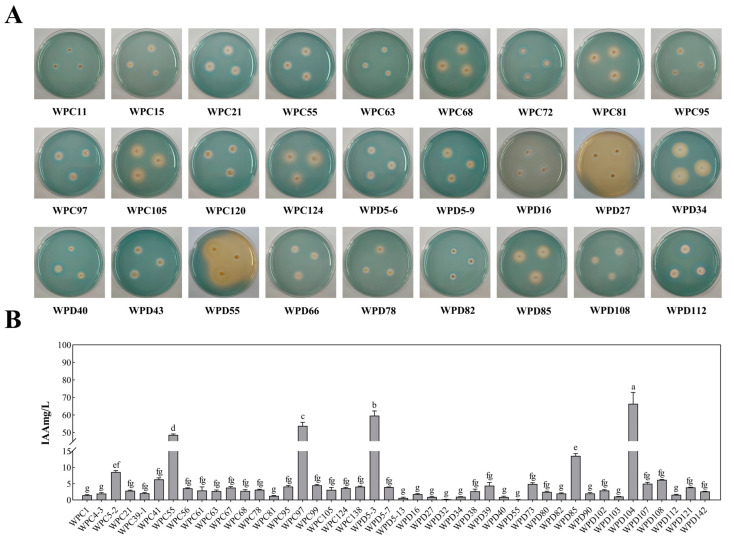
The growth-promoting ability of the rescreened strains. (**A**) Iron carrier production capacity of some strains. (**B**) IAA production capacity of some strains. Each experiment was performed with at least three biological replicates. Different lowercase letters indicate significant differences between treatments (*p* < 0.05).

A total of 51 rescreened PSBs were analyzed for iron carrier production and IAA-producing capacity to evaluate their plant growth-promoting potential, given the critical roles of auxin and iron in plant development. As shown in Figure 3A, siderophore production capacity was visualized through orange halo formation, with larger halos indicating stronger activity. Strains WPD16, WPD27, WPD55, WPD34, and WPC105 exhibited prominent iron carrier production. Similarly, IAA biosynthesis capacity was assessed (Figure 3B), revealing high performance in strains WPD104, WPD5-3, WPC97, WPC55, and WPD85. Notably, several strains demonstrated dual capabilities, excelling in both phosphate solubilization and growth-promoting functions.

### 3.3. Screening and Physiological and Biochemical Characterization of the Dominant Strains

In order to obtain more outstanding phosphate-solubilizing capacity strains, eight dominant PSB strains were screened again for two kinds of phosphate-solubilizing capacity, as well as prophylactic capacity assay: WPD34, WPD103, WPD24, WPD16, WPD5-1, WPD5-9, WPD85, and WPC99. The morphology characteristics of the eight dominant PSB strains were observed and recorded (Table 1). Most of the phosphate-solubilizing bacteria were round and opaque, had a folded surface, were yellow in color, and had moist colonies. A few showed irregular shape, smooth surface, white color, and drier colonies.

**Figure 4:**
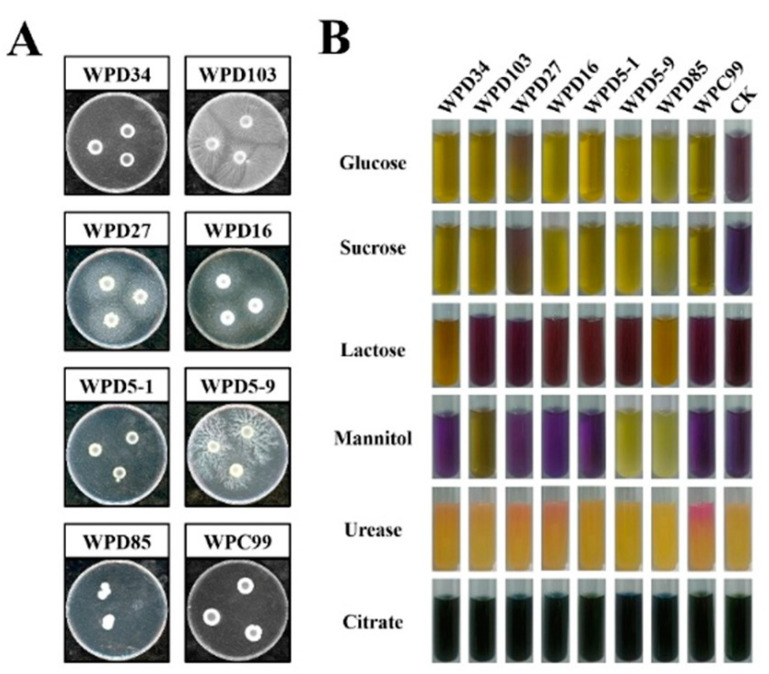
Diagrams of some physiological and biochemical characteristics of the eight dominant strains. (**A**) Lipase medium. (**B**) Sugar fermentation, urease, and citrate utilization tests.

The various physiological and biochemical characterizations of the eight strains are shown in Table 2 and Figure 4. WPD103, WPD27, WPD16, and WPD5-9 were able to produce lipase, while WPC99 had a significant ability to produce urease. All eight strains were able to utilize glucose and sucrose, and they were only partially able to utilize lactose and mannitol. Among them, WPD27, WPD16, WPD5-9, and WPD85 were also able to utilize citrate. These physiological and biochemical characterizations will be useful for further metabolic studies of the strains.

The phylogenetic tree of PSB strains identified based on 16S rRNA sequences is shown in Figure 2. The 16S rRNA sequences of the eight dominant PSB strains were highly similar to those of known bacteria in the genera *Bacillus*, *Burkholderia*, *Pantoea*, and *Paenibacillus*. These strains may represent new species, and taxonomic studies are in progress.

### 3.4. Biofilm Formation Capacity of Dominant Strains

**Figure 5:**
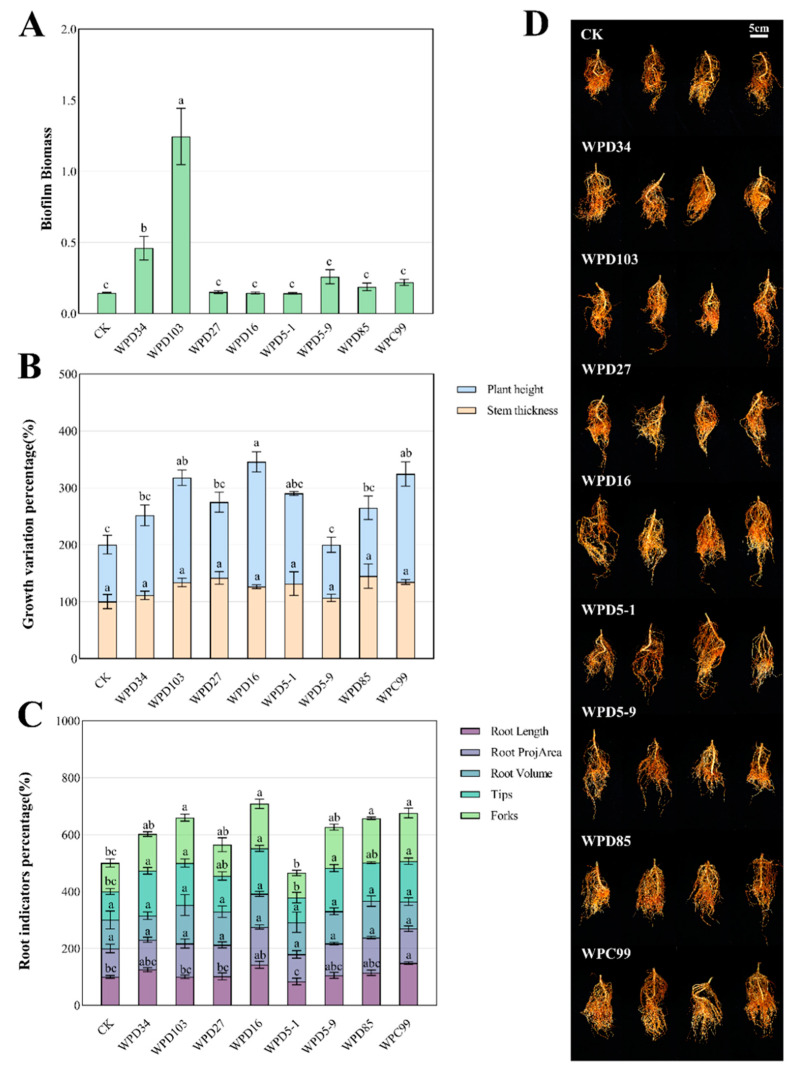
Biofilm formation capacity of eight dominant strains and potting experiment. (**A**) Biofilm formation capacity. (**B**) Percentage change in growth of peach seedlings. (**C**) Percentage of root system indexes (root length, root ProjArea, root volume, tips, and forks) in peach seedlings. (**D**) Picture of peach seedling root system after 40 days of treatment. Each experiment was performed with at least three biological replications. Different lowercase letters indicate significant differences between treatments (*p* < 0.05).

In order to investigate the resistance defense of the strains, the biofilm formation capacity was determined. WPD103 had the strongest biofilm formation ability, which was significantly higher than that of the control group and other treatment groups, reaching 1.09, and followed by WPD34, reaching 0.52 (Figure 5A). It indicates that WPD1013 and WPD34 have a stronger resistance defense ability compared with other groups, and their bacterial communities are more stable. The related content about the biofilm-resistance defense mechanism deserves further study.

### 3.5. Potting Trials

In order to verify the pairwise strain-related effects of the dominant strains, pot experiments were conducted. Peach seedlings were treated with different bacterial suspensions of eight dominant strains. Then, the amount of change in plant height and stem thickness before and after treatment was measured, and the root conformation of peach seedlings was analyzed to calculate the percentage of each index relative to the control in different treatment groups (Figure 5B,C). The pictures of the root system after 40 days of treatment are shown in Figure 5D. Compared with CK, the amount of change in plant height, root length, tips, and forks of peach seedlings was significantly increased by WPC99 treatment; the amount of change in plant height, tips, and forks of peach seedlings was significantly increased by WPD16 treatment; and the amount of change in plant height, tips, and forks of peach seedlings was significantly increased by WPD103 treatment. Only the tips were significantly increased by WPD5-9 treatment, and only the forks were significantly increased by WPD85 treatment. The combined effect of WPD16 on the growth of peach seedlings was the strongest among increasing plant height change by 119% compared to the control (*p* < 0.05), indicating that WPD16 could significantly promote the growth of plants.

## 4. Discussion

### 4.1. Isolation of PSBs

Since the 1950s, domestic and international research on PSBs and their applications has progressively advanced. The plate method, as the most prevalent isolation technique for PSBs, demonstrates broad applicability across various ecosystems. The Pikovskaya (PVK) medium has been predominantly employed for initial screening of these microorganisms. Subsequent studies have optimized the nutritional composition of culture media, leading to the development of improved screening protocols. In 1999, the National Botanical Research Institute introduced a novel phosphate growth medium (NBRIP). Comparative analyses revealed that NBRIP exhibits superior efficacy in screening PSBs compared to PVK, particularly in broth culture assays where NBRIP consistently demonstrated approximately threefold higher efficiency than PVK [21]. In the current investigation, the isolation and screening of PSBs were conducted using PVK medium through plate culture methodology. This involved soil incubation, followed by uniform plate inoculation and subsequent purification of individual colonies to obtain axenic phosphate-solubilizing cultures. Quantitative assessment of phosphate-solubilizing capacity was performed using NBRIP medium. The methodological framework adopted in this study therefore ensures reliable isolation and characterization of PSBs.

Extensive research findings have identified *Bacillus* and *Pseudomonas* as predominant PSBs [30,31,32,33,34,35]. The current study successfully isolated 20 strains of *Bacillus* and 4 strains of *Pseudomonas*, aligning with established research paradigms. Notably, additional strains from ten other genera were additionally identified through systematic screening. This greatly enriches the resource base of PSBs. Although prior studies have documented phosphate-solubilizing capabilities within some PSBs, the specific strains isolated in this investigation exhibit unique phylogenetic characteristics, as evidenced by genomic database analyses where no identical sequences were retrievable from existing repositories. Consequently, these novel isolates not only expand the documented diversity of PSBs but also provide valuable microbial resources for developing specialized biofertilizers tailored to fruit tree cultivation. This discovery specifically enriches the repository of PSBs derived from arboreal ecosystems and advances the strategic development of microbial consortia optimized for pomological applications.

In this study, strains exhibiting tricalcium phosphate (Ca_3_(PO_4_)_2_) solubilization were screened using PKV and NBRIP media. However, it should be noted that the specificity of these media imposes limitations on the generalizability of our conclusions. Both PKV and NBRIP employ Ca_3_(PO_4_)_2_ as the sole insoluble phosphorus source, meaning the results solely demonstrate the strains’ capacity to solubilize Ca_3_(PO_4_)_2_, with no direct evidence for their efficacy against other insoluble phosphates, such as FePO_4_, AlPO_4_, or organic phosphorus compounds. This constraint likely stems from mechanistic differences in phosphate solubilization: Ca_3_(PO_4_)_2_ dissolution primarily relies on pH reduction via organic acid secretion, whereas FePO_4_ or AlPO_4_ solubilization may involve chelation, enzymatic hydrolysis, or specialized transport systems. Consequently, future studies should employ phosphate-specific media (e.g., FePO_4_ as the sole P source) or integrate molecular approaches (e.g., analysis of acid phosphatase gene clusters) to comprehensively evaluate the multifunctional phosphate-solubilizing potential of these strains. Despite this limitation, our findings provide a foundational framework for developing Ca_3_(PO_4_)_2_-targeted biofertilizers, while underscoring the necessity to optimize strain adaptability to diverse phosphorus sources in subsequent applications.

### 4.2. Phosphate-Solubilizing Capacity of the Strains

Microorganisms capable of forming halo zones were selected as potential phosphate solubilizers [36]. The presence of halo zones served as one of the screening criteria for PSBs, with the halo size being used to determine the phosphate solubilization index and considered an indicator for evaluating bacterial phosphate-solubilizing capacity. Simultaneously, the phosphate-solubilizing capacity of strains in liquid media was also adopted as a crucial evaluation index. This study measured both identification indicators of phosphate-solubilizing capacity, revealing that most strains with high phosphate solubilization index did not exhibit correspondingly high soluble phosphorus content. The discrepancy between phosphate solubilization index and soluble phosphorus content may arise from differential expression of organic acids or phosphatases under varying culture conditions. This indicates poor correlation between soluble phosphorus content in inoculated media and phosphate solubilization index, which is insufficient for quantifying the solubilization capacity of PSB. These findings align with previous research results [33]. However, the differential environmental conditions experienced by strains in liquid versus solid media may lead to distinct phosphate-solubilization mechanisms, potentially accounting for the observed discrepancies between these two evaluation indicators. Nevertheless, the underlying principles require more in-depth investigation. Notably, among the eight dominant strains examined, WPD5-9 demonstrated a strong correlation between phosphate solubilization index and soluble phosphorus content, both showing significantly enhanced values. This suggests that WPD5-9 may employ identical phosphate-solubilization strategies in different media or be influenced by specific common substances, although its operational mechanisms warrant further exploration.

Given the generally weak correlation between halo size and soluble phosphorus content observed in most cases, the quantification of phosphate-solubilizing capacity in PSBs has predominantly relied on soluble phosphorus content measurements in previous studies [37]. Some research has demonstrated that selected PSBs can achieve soluble phosphorus levels up to 100 mg/L [33,38]. However, the soluble phosphorus content of PSBs identified in this study ranged only between 3 and 20 mg/L. This discrepancy may be attributed to the smaller culture medium volume and prolonged incubation period, during which PSBs might have completely solubilized the insoluble phosphate in the medium. Concurrently, PSBs could assimilate soluble phosphorus from the solution to sustain normal cellular metabolic activities, thereby reducing the soluble phosphorus content in the medium. A critical limitation of using supernatant-soluble phosphorus concentration as the sole screening parameter lies in its failure to account for cellular phosphorus utilization. Therefore, to achieve more objective evaluation of in vitro phosphate-solubilizing capacity, it is essential to appropriately select culture medium volume and incubation duration, while simultaneously determining the cellular phosphorus content of PSB strains [35].

### 4.3. Prospects for the Application of PSBs

Despite the critical role of phosphorus fertilizers in ensuring crop growth and agricultural productivity, their low utilization efficiency and persistent application have generated significant environmental concerns [39,40]. PSBs are increasingly recognized as an environmentally sustainable and cost-effective alternative to chemical phosphorus fertilizers [41,42]. Beyond enhancing plant phosphorus uptake, PSBs demonstrate ecological benefits including remediation of soil contaminated by conventional fertilizers, improvement of soil microbial community diversity and abundance [4,43,44,45], mitigation of heavy metal toxicity in plants, and suppression of fungal pathogen invasions [46,47,48,49,50,51]. These multifunctional attributes position PSBs as a highly promising microbial fertilizer resource, garnering substantial research interest for their potential to advance sustainable agricultural practices.

The exploration of novel PSB strains holds significant scientific and practical value. This study identified multiple functionally distinct PSBs isolated from fruit trees, exhibiting not only phosphate-solubilizing capacity but also iron carrier production, IAA biosynthesis, and biofilm-formation capabilities. Strains demonstrating such exceptional traits qualify as candidates for microbial fertilizer development [6]. To validate their agricultural applicability, eight dominant PSBs were subjected to pot experiments. Notably, *Burkholderia* sp. WPD16 exhibited superior phosphate solubilization, iron carrier production, and statistically significant plant growth promotion. These findings align with previous reports on plant growth-promoting rhizobacteria (PGPRs) within the *Burkholderia* genus [52,53], while its siderophore-mediated growth enhancement corroborates established mechanisms [54,55]. Unlike previous studies focusing on *Bacillus* spp., we identified *Burkholderia* sp. WPD16 as a novel PSB with dual functionalities, expanding the taxonomic diversity of agriculturally relevant PSBs. Future field trials should validate WPD16′s efficacy under orchard conditions and explore its synergy with other PGPR strains. Collectively, WPD16 particularly holds promise for the development of specialized microbial fertilizers tailored for fruit tree cultivation. Its implementation could significantly contribute to advancing agricultural sustainability through developing ecosystem-specific biofertilizer formulation, enhancing nutrient use efficiency in perennial crops, and reducing dependence on chemical fertilizer. It is worth noting that the agricultural application of *Burkholderia* strains faces challenges, including biosafety concerns (pathogenicity risks and horizontal gene transfer), environmental adaptability limitations, industrialization barriers (fermentation costs and formulation stability), undefined ecological impacts, and market acceptance thresholds. Despite these constraints, precedents like *Burkholderia phytofirmans* PsJN demonstrate their potential, warranting targeted studies on virulence attenuation, multi-stress-resilience engineering, and ecological risk assessment to advance WPD16 toward scalable biofertilizer development.

While the 40-day pot experiment demonstrated the short-term efficacy of strain WPD16 in enhancing peach seedling growth (119% height increment), these findings must be interpreted within the constraints of controlled environments. First, the limited experimental duration precludes assessment of sustained microbial colonization or delayed ecological impacts, such as potential shifts in rhizosphere microbiota composition over multiple growth cycles. Second, greenhouse conditions inherently lack field-level stressors, like diurnal temperature fluctuations, precipitation variability, and interspecies competition, which critically influence microbial survival and functionality [56]. Third, the single growth stage (seedling phase) evaluated here does not reflect the dynamic nutrient demands of peach trees during flowering and fruit maturation. To bridge this gap, future studies should implement 2–3-year field trials across distinct phenological stages, integrating soil metagenomic profiling and nutrient flux analysis. Parallel investigations on microbial persistence under region-specific abiotic stresses (e.g., seasonal droughts in Shandong peach orchards) will further contextualize WPD16′s agricultural applicability.

Critical considerations arise regarding microbial fertilizer application strategies. Current practices typically involve introducing strains from foreign ecosystems into new environments, yet the efficacy of reintroducing native strains into their original ecosystems remains underexplored. Furthermore, comparative analyses of single-strain versus consortium-based treatments warrant systematic investigation. These knowledge gaps highlight the necessity for targeted research to optimize microbial fertilizer design. Ultimately, the advancement of scientifically informed, ecosystem-specific microbial fertilizers represents an indispensable pathway toward achieving agricultural sustainability.

## 5. Conclusions

This study isolated 51 high-efficiency phosphate-solubilizing bacteria (PSBs) from peach rhizospheres, dominated by *Bacillus* and *Burkholderia*, enriching the taxonomic diversity of fruit tree-associated PSBs. *Burkholderia* sp. WPD16 emerged as a multifunctional candidate, demonstrating exceptional inorganic phosphate solubilization (D/d = 2.99) and iron carrier production, and enhancing peach seedling height by 119% in pot experiments. To advance WPD16 toward agricultural application, future work must prioritize multi-year field trials across peach phenological stages, biosafety optimization via virulence gene editing, and formulation development for agricultural applications. Synergistic consortium development with complementary PGPR strains could further enhance nutrient-use efficiency. These findings position WPD16 as a pivotal resource for reducing chemical fertilizer dependency in orchard ecosystems through microbiome-driven strategies.

## Figures and Tables

**Table 1:** Colony morphology of PBS from the rhizosphere soil of peach trees.

Strains	Shape	Verge	Colony Morphology	Dryness	Transparency	Color
WPD34	Orbicular	Undulate	Wrinkled	Dry	Opaque	Bright yellow
WPD103	Orbicular	Undulate	Wrinkled	Moist	Opaque	Bright yellow
WPD27	Orbicular	Neatly	Smooth	Moist	Opaque	Earthy yellow
WPD16	Orbicular	Neatly	Smooth	Moist	Opaque	Yellow
WPD5-1	Orbicular	Undulate	Smooth	Moist	Opaque	Bright yellow
WPD5-9	Orbicular	Undulate	Wrinkled	Moist	Opaque	Light yellow
WPD85	Irregular	Undulate	Wrinkled	Moist	Transparent	White
WPC99	Orbicular	Undulate	Wrinkled	Dry	Opaque	Milky white

**Table 2:** Phylogenetic classification, and physiological and biochemical characteristics of 8 PSBs.

Strains	Genus	1	2	3	4	5	6	7	8	9	10	11	12	13
WPD34	*Bacillus* sp.	+	+	+	−	−	−	+	+	+	−	−	−	−
WPD103	*Bacillus* sp.	+	+	−	+	−	+	−	+	+	−	−	−	−
WPD27	*Burkholderia* sp.	+	+	−	+	−	+	+	−	−	+	+	+	+
WPD16	*Burkholderia* sp.	+	+	+	+	−	+	+	−	−	+	+	+	+
WPD5-1	*Pantoea* sp.	+	+	+	−	−	−	−	+	+	−	+	+	+
WPD5-9	*Pantoea* sp.	+	+	+	+	−	+	−	+	+	+	−	−	+
WPD85	*Paenibacillus* sp.	+	+	+	+	−	−	−	+	+	+	+	−	+
WPC99	*Bacillus* sp.	+	+	−	−	+	−	+	+	+	−	−	−	+

Note: “+” means a positive reaction; “−” means a negative reaction. The physiological and biochemical indicators 1–13 are glucose, sucrose, lactose, mannitol, starch hydrolysis, lipase, urease, M.R., catalase, citrate utilization, kana, rifampicin, and ampicillin.

## Data Availability

Nucleotide sequences were deposited in the GenBank database under the accession nos. PV273821-PV273871. The data that support the findings of this study are available from the authors upon request.

## Supplementary Materials

The following supporting information can be downloaded at: https://www.mdpi.com/article/10.3390/microorganisms13040718/s1, File S1: Methods of physiological and biochemical experiments.
