# Supplementary material for: Isolation, Characterization and Growth-Promoting Properties of Phosphate-Solubilizing Bacteria (PSBs) Derived from Peach Tree Rhizosphere
Source: Microorganisms. 2025 Mar 23;13(4):718. doi: 10.3390/microorganisms13040718 (PMC12029301; doi:10.3390/microorganisms13040718)
Supplement: Supplementary file 1 [file microorganisms-13-00718-s001.zip › microorganisms-3508494-supplementary.pdf]

Methods of physiological and biochemical experiments

1. Glycolysis Test

Inoculate three tubes each of glucose, sucrose, lactose, and mannitol media per strain, leaving one uninoculated tube as control. Incubate at 37°C for 24-72 hr. Positive results are indicated by media decolorization or pale-yellow coloration; negative results retain original color.

Medium: 5.0g peptone, 3.0g beef extract, 1% test carbohydrate, 1mL 1.6% bromocresol purple, 1000mL H_2O (pH 7.2-7.5). Dissolve ingredients, add indicator, sterilize at 115°C for 20 min.

2. Starch Hydrolysis Test

Streak cultures on starch-beef extract agar plates. After 2-4 days at 37°C, flood with iodine solution. Clear zones around colonies indicate starch hydrolysis (positive).

Medium: 20g soluble starch, 3g beef extract, 1.8g agar, 1000mL H_2O (pH 7.2-7.4). Sterilize at 121°C for 20 min.

3. Catalase Test

Transfer colonies to a slide, add 3% H_2O_2. Immediate bubble formation indicates catalase activity (positive).

4. Methyl Red (MR) Test

Inoculate glucose-peptone broth, incubate 48 hr at 37°C. Add 3-4 drops methyl red: red coloration (pH $\leq$4.4) = positive; yellow = negative.

Medium: 5g KH_2PO_4, 5g glucose, 7g peptone, 1000mL H_2O (pH 7.0). Sterilize at 115°C for 30 min.

5. Citrate Utilization Test

Inoculate Simmons' citrate agar slants. Positive results show bacterial growth with color change from green to blue after 4 days at 37°C.

Medium: 5g NaCl, 1g $K_2HPO_4 \cdot 3H_2O$, 1g $(NH_4)H_2PO_4$, 0.2g $MgSO_4 \cdot 7H_2O$, 1g sodium citrate, 10mL 1% bromothymol blue, 990mL H_2O (pH 7.0). Sterilize at 121°C for 15 min.

6. Antibiotic Susceptibility Test

Spread bacterial suspension uniformly on agar. Apply antibiotic-impregnated discs. Measure inhibition zones after 24-48 h incubation at 30°C.

7. Lipase Test

Spot-inoculate lipase agar. Observe daily for 7 days. Positive: opaque halo around colonies.

Medium: 10g peptone, 5g NaCl, 0.1g $CaCl_2 \cdot 7H_2O$, 9g agar, 1000mL H_2O (pH 7.4). Add sterile Tween 80 to 1% final concentration post-sterilization.

8. Urease Test

Streak cultures on urea agar slants. Positive: pink-red coloration within 4 days.

Medium: 1g peptone, 5g NaCl, 1g glucose, 2g KH_2PO_4, 6mL 0.2% phenol red, 20g agar, 1000mL H_2O (pH 6.8-6.9). Add filter-sterilized 20% urea to 2% final concentration post-sterilization.
